# Supplementary figures and images for: Field pea leaf disease classification using a deep learning approach (part 2 of 2)
Source: PLoS One. 2024 Jul 25;19(7):e0307747. doi: 10.1371/journal.pone.0307747 (PMC11271925; doi:10.1371/journal.pone.0307747)

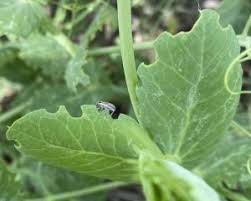

Supplement: S1 Dataset — (ZIP) [file pone.0307747.s001.zip › field pea dataset/Testing/Field_Pea_Healthy/Healthy 1011 (55).jpg]

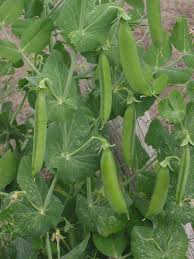

Supplement: S1 Dataset — (ZIP) [file pone.0307747.s001.zip › field pea dataset/Testing/Field_Pea_Healthy/Healthy 1011 (56).jpg]

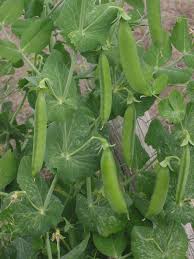

Supplement: S1 Dataset — (ZIP) [file pone.0307747.s001.zip › field pea dataset/Testing/Field_Pea_Healthy/Healthy 1011 (57).jpg]

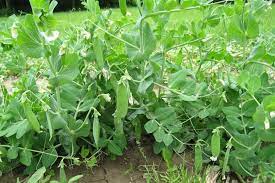

Supplement: S1 Dataset — (ZIP) [file pone.0307747.s001.zip › field pea dataset/Testing/Field_Pea_Healthy/Healthy 1011 (58).jpg]

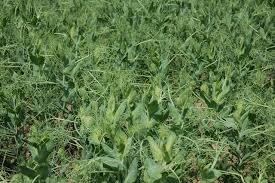

Supplement: S1 Dataset — (ZIP) [file pone.0307747.s001.zip › field pea dataset/Testing/Field_Pea_Healthy/Healthy 1011 (59).jpg]

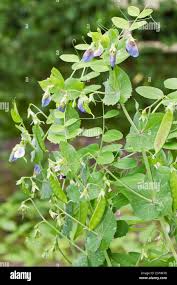

Supplement: S1 Dataset — (ZIP) [file pone.0307747.s001.zip › field pea dataset/Testing/Field_Pea_Healthy/Healthy 1011 (6).jpg]

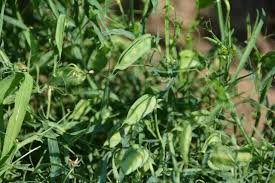

Supplement: S1 Dataset — (ZIP) [file pone.0307747.s001.zip › field pea dataset/Testing/Field_Pea_Healthy/Healthy 1011 (60).jpg]

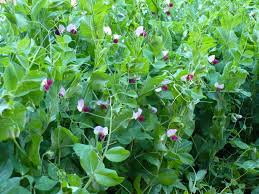

Supplement: S1 Dataset — (ZIP) [file pone.0307747.s001.zip › field pea dataset/Testing/Field_Pea_Healthy/Healthy 1011 (7).jpg]

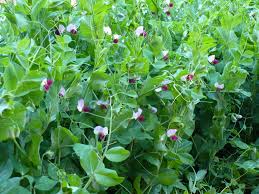

Supplement: S1 Dataset — (ZIP) [file pone.0307747.s001.zip › field pea dataset/Testing/Field_Pea_Healthy/Healthy 1011 (8).jpg]

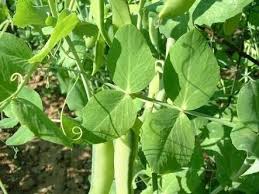

Supplement: S1 Dataset — (ZIP) [file pone.0307747.s001.zip › field pea dataset/Testing/Field_Pea_Healthy/Healthy 1011 (9).jpg]

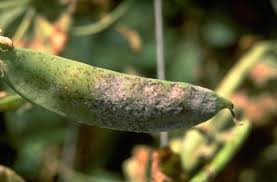

Supplement: S1 Dataset — (ZIP) [file pone.0307747.s001.zip › field pea dataset/Testing/Field_Pea_Leaf_Spot/leaf spot11 101 (1).jpg]

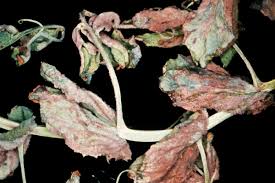

Supplement: S1 Dataset — (ZIP) [file pone.0307747.s001.zip › field pea dataset/Testing/Field_Pea_Leaf_Spot/leaf spot11 101 (2).jpg]

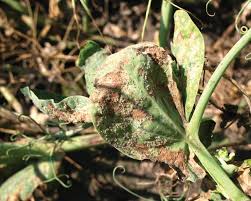

Supplement: S1 Dataset — (ZIP) [file pone.0307747.s001.zip › field pea dataset/Testing/Field_Pea_Leaf_Spot/leaf spot11 101 (4).jpg]

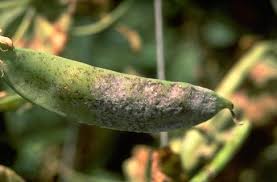

Supplement: S1 Dataset — (ZIP) [file pone.0307747.s001.zip › field pea dataset/Testing/Field_Pea_Leaf_Spot/leaf spot111 (1).jpg]

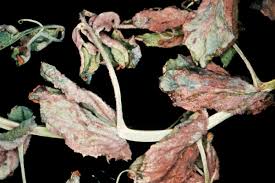

Supplement: S1 Dataset — (ZIP) [file pone.0307747.s001.zip › field pea dataset/Testing/Field_Pea_Leaf_Spot/leaf spot111 (10).jpg]

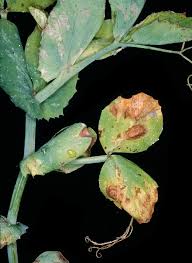

Supplement: S1 Dataset — (ZIP) [file pone.0307747.s001.zip › field pea dataset/Testing/Field_Pea_Leaf_Spot/leaf spot111 (11).jpg]

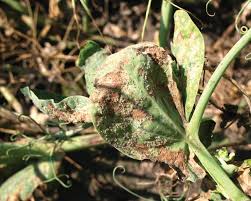

Supplement: S1 Dataset — (ZIP) [file pone.0307747.s001.zip › field pea dataset/Testing/Field_Pea_Leaf_Spot/leaf spot111 (15).jpg]

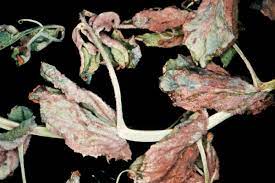

Supplement: S1 Dataset — (ZIP) [file pone.0307747.s001.zip › field pea dataset/Testing/Field_Pea_Leaf_Spot/leaf spot111 (17).jpg]

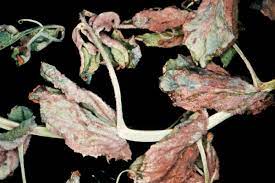

Supplement: S1 Dataset — (ZIP) [file pone.0307747.s001.zip › field pea dataset/Testing/Field_Pea_Leaf_Spot/leaf spot111 (19).jpg]

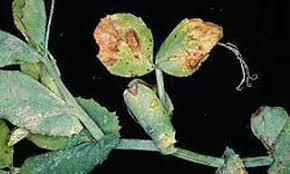

Supplement: S1 Dataset — (ZIP) [file pone.0307747.s001.zip › field pea dataset/Testing/Field_Pea_Leaf_Spot/leaf spot111 (21).jpg]

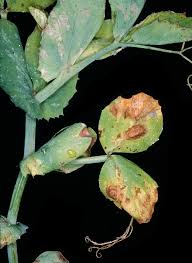

Supplement: S1 Dataset — (ZIP) [file pone.0307747.s001.zip › field pea dataset/Testing/Field_Pea_Leaf_Spot/leaf spot111 (23).jpg]

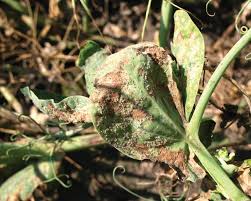

Supplement: S1 Dataset — (ZIP) [file pone.0307747.s001.zip › field pea dataset/Testing/Field_Pea_Leaf_Spot/leaf spot111 (27).jpg]

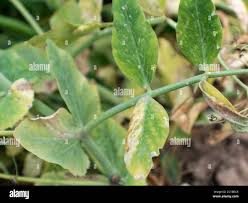

Supplement: S1 Dataset — (ZIP) [file pone.0307747.s001.zip › field pea dataset/Testing/Field_Pea_Leaf_Spot/leaf spot111 (37).jpg]

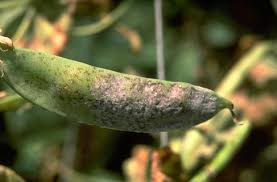

Supplement: S1 Dataset — (ZIP) [file pone.0307747.s001.zip › field pea dataset/Testing/Field_Pea_Leaf_Spot/leaf spot111 (39).jpg]

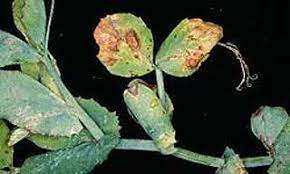

Supplement: S1 Dataset — (ZIP) [file pone.0307747.s001.zip › field pea dataset/Testing/Field_Pea_Leaf_Spot/leaf spot111 (43).jpg]

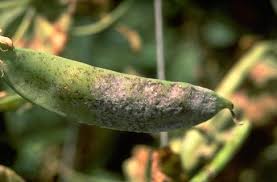

Supplement: S1 Dataset — (ZIP) [file pone.0307747.s001.zip › field pea dataset/Testing/Field_Pea_Leaf_Spot/leaf spot111 (49).jpg]

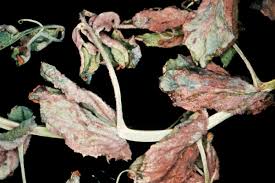

Supplement: S1 Dataset — (ZIP) [file pone.0307747.s001.zip › field pea dataset/Testing/Field_Pea_Leaf_Spot/leaf spot111 (50).jpg]

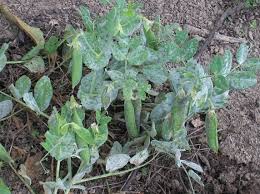

Supplement: S1 Dataset — (ZIP) [file pone.0307747.s001.zip › field pea dataset/Testing/Field_Pea_Powdery_Mildew/powdery_mildew (10).jpg]

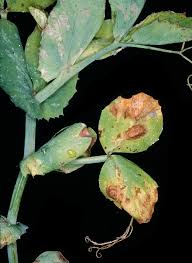

Supplement: S1 Dataset — (ZIP) [file pone.0307747.s001.zip › field pea dataset/Testing/Field_Pea_Powdery_Mildew/powdery_mildew (11).jpg]

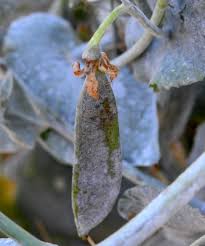

Supplement: S1 Dataset — (ZIP) [file pone.0307747.s001.zip › field pea dataset/Testing/Field_Pea_Powdery_Mildew/powdery_mildew (12).jpg]

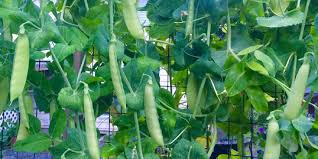

Supplement: S1 Dataset — (ZIP) [file pone.0307747.s001.zip › field pea dataset/Testing/Field_Pea_Powdery_Mildew/powdery_mildew (14).jpg]

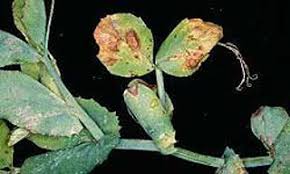

Supplement: S1 Dataset — (ZIP) [file pone.0307747.s001.zip › field pea dataset/Testing/Field_Pea_Powdery_Mildew/powdery_mildew (15).jpg]

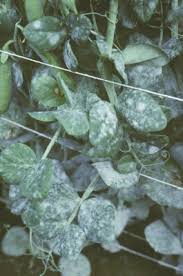

Supplement: S1 Dataset — (ZIP) [file pone.0307747.s001.zip › field pea dataset/Testing/Field_Pea_Powdery_Mildew/powdery_mildew (16).jpg]

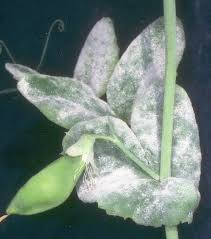

Supplement: S1 Dataset — (ZIP) [file pone.0307747.s001.zip › field pea dataset/Testing/Field_Pea_Powdery_Mildew/powdery_mildew (17).jpg]

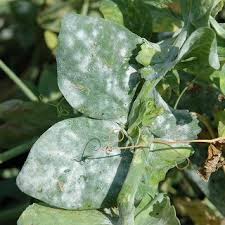

Supplement: S1 Dataset — (ZIP) [file pone.0307747.s001.zip › field pea dataset/Testing/Field_Pea_Powdery_Mildew/powdery_mildew (18).jpg]

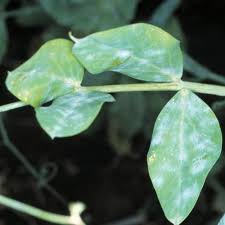

Supplement: S1 Dataset — (ZIP) [file pone.0307747.s001.zip › field pea dataset/Testing/Field_Pea_Powdery_Mildew/powdery_mildew (19).jpg]

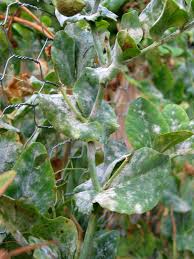

Supplement: S1 Dataset — (ZIP) [file pone.0307747.s001.zip › field pea dataset/Testing/Field_Pea_Powdery_Mildew/powdery_mildew (2).jpg]

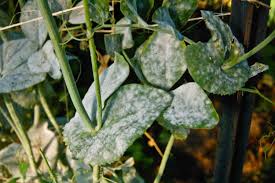

Supplement: S1 Dataset — (ZIP) [file pone.0307747.s001.zip › field pea dataset/Testing/Field_Pea_Powdery_Mildew/powdery_mildew (20).jpg]

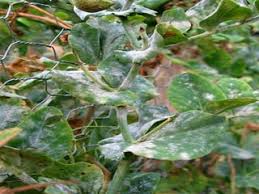

Supplement: S1 Dataset — (ZIP) [file pone.0307747.s001.zip › field pea dataset/Testing/Field_Pea_Powdery_Mildew/powdery_mildew (21).jpg]

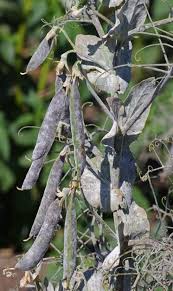

Supplement: S1 Dataset — (ZIP) [file pone.0307747.s001.zip › field pea dataset/Testing/Field_Pea_Powdery_Mildew/powdery_mildew (3).jpg]

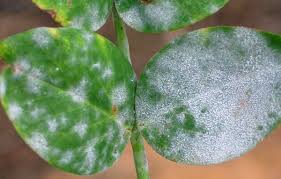

Supplement: S1 Dataset — (ZIP) [file pone.0307747.s001.zip › field pea dataset/Testing/Field_Pea_Powdery_Mildew/powdery_mildew (4).jpg]

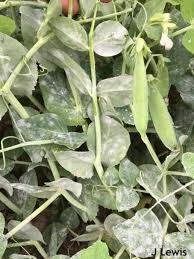

Supplement: S1 Dataset — (ZIP) [file pone.0307747.s001.zip › field pea dataset/Testing/Field_Pea_Powdery_Mildew/powdery_mildew (5).jpg]

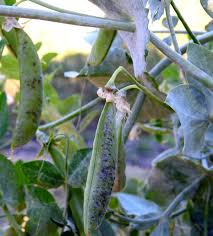

Supplement: S1 Dataset — (ZIP) [file pone.0307747.s001.zip › field pea dataset/Testing/Field_Pea_Powdery_Mildew/powdery_mildew (6).jpg]

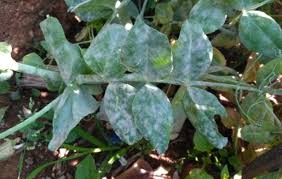

Supplement: S1 Dataset — (ZIP) [file pone.0307747.s001.zip › field pea dataset/Testing/Field_Pea_Powdery_Mildew/powdery_mildew (7).jpg]

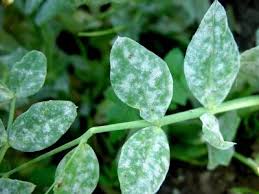

Supplement: S1 Dataset — (ZIP) [file pone.0307747.s001.zip › field pea dataset/Testing/Field_Pea_Powdery_Mildew/powdery_mildew (8).jpg]
