# Supplementary figures and images for: Field pea leaf disease classification using a deep learning approach (part 1 of 2)
Source: PLoS One. 2024 Jul 25;19(7):e0307747. doi: 10.1371/journal.pone.0307747 (PMC11271925; doi:10.1371/journal.pone.0307747)

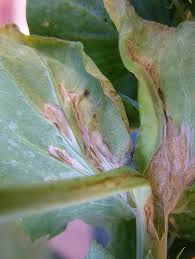

Supplement: S1 Dataset — (ZIP) [file pone.0307747.s001.zip › field pea dataset/Testing/Field_Pea_Ascochyta_Blight/Blight 30 (1).jpg]

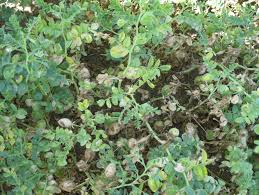

Supplement: S1 Dataset — (ZIP) [file pone.0307747.s001.zip › field pea dataset/Testing/Field_Pea_Ascochyta_Blight/Blight 30 (10).jpg]

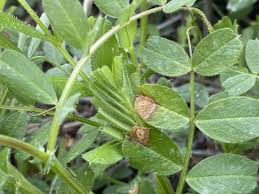

Supplement: S1 Dataset — (ZIP) [file pone.0307747.s001.zip › field pea dataset/Testing/Field_Pea_Ascochyta_Blight/Blight 30 (11).jpg]

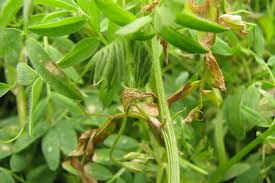

Supplement: S1 Dataset — (ZIP) [file pone.0307747.s001.zip › field pea dataset/Testing/Field_Pea_Ascochyta_Blight/Blight 30 (12).jpg]

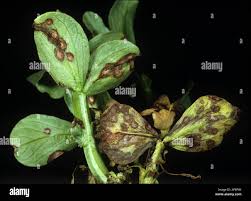

Supplement: S1 Dataset — (ZIP) [file pone.0307747.s001.zip › field pea dataset/Testing/Field_Pea_Ascochyta_Blight/Blight 30 (13).jpg]

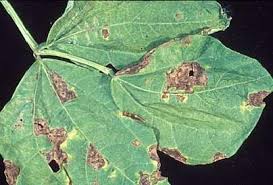

Supplement: S1 Dataset — (ZIP) [file pone.0307747.s001.zip › field pea dataset/Testing/Field_Pea_Ascochyta_Blight/Blight 30 (14).jpg]

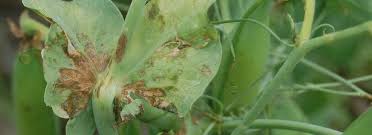

Supplement: S1 Dataset — (ZIP) [file pone.0307747.s001.zip › field pea dataset/Testing/Field_Pea_Ascochyta_Blight/Blight 30 (15).jpg]

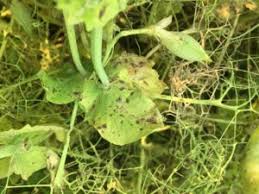

Supplement: S1 Dataset — (ZIP) [file pone.0307747.s001.zip › field pea dataset/Testing/Field_Pea_Ascochyta_Blight/Blight 30 (16).jpg]

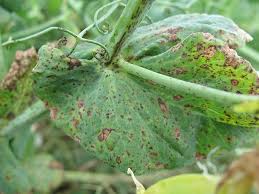

Supplement: S1 Dataset — (ZIP) [file pone.0307747.s001.zip › field pea dataset/Testing/Field_Pea_Ascochyta_Blight/Blight 30 (17).jpg]

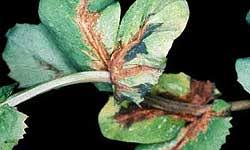

Supplement: S1 Dataset — (ZIP) [file pone.0307747.s001.zip › field pea dataset/Testing/Field_Pea_Ascochyta_Blight/Blight 30 (18).jpg]

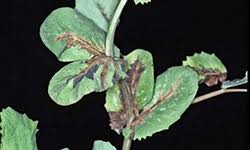

Supplement: S1 Dataset — (ZIP) [file pone.0307747.s001.zip › field pea dataset/Testing/Field_Pea_Ascochyta_Blight/Blight 30 (19).jpg]

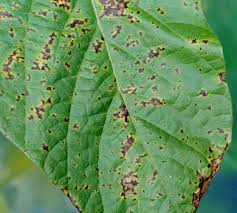

Supplement: S1 Dataset — (ZIP) [file pone.0307747.s001.zip › field pea dataset/Testing/Field_Pea_Ascochyta_Blight/Blight 30 (2).jpg]

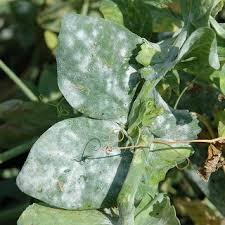

Supplement: S1 Dataset — (ZIP) [file pone.0307747.s001.zip › field pea dataset/Testing/Field_Pea_Ascochyta_Blight/Blight 30 (20).jpg]

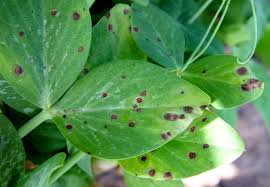

Supplement: S1 Dataset — (ZIP) [file pone.0307747.s001.zip › field pea dataset/Testing/Field_Pea_Ascochyta_Blight/Blight 30 (21).jpg]

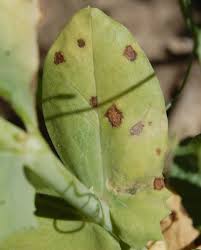

Supplement: S1 Dataset — (ZIP) [file pone.0307747.s001.zip › field pea dataset/Testing/Field_Pea_Ascochyta_Blight/Blight 30 (22).jpg]

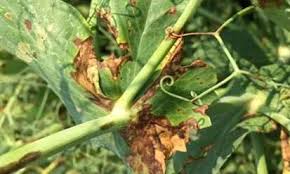

Supplement: S1 Dataset — (ZIP) [file pone.0307747.s001.zip › field pea dataset/Testing/Field_Pea_Ascochyta_Blight/Blight 30 (23).jpg]

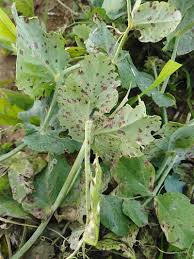

Supplement: S1 Dataset — (ZIP) [file pone.0307747.s001.zip › field pea dataset/Testing/Field_Pea_Ascochyta_Blight/Blight 30 (24).jpg]

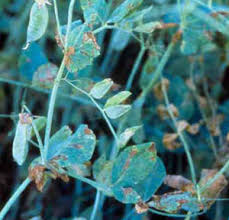

Supplement: S1 Dataset — (ZIP) [file pone.0307747.s001.zip › field pea dataset/Testing/Field_Pea_Ascochyta_Blight/Blight 30 (25).jpg]

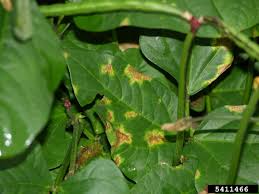

Supplement: S1 Dataset — (ZIP) [file pone.0307747.s001.zip › field pea dataset/Testing/Field_Pea_Ascochyta_Blight/Blight 30 (26).jpg]

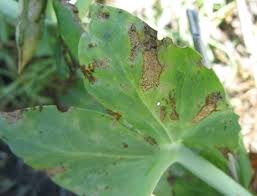

Supplement: S1 Dataset — (ZIP) [file pone.0307747.s001.zip › field pea dataset/Testing/Field_Pea_Ascochyta_Blight/Blight 30 (27).jpg]

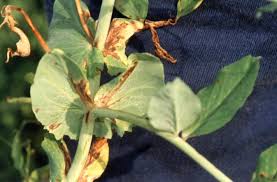

Supplement: S1 Dataset — (ZIP) [file pone.0307747.s001.zip › field pea dataset/Testing/Field_Pea_Ascochyta_Blight/Blight 30 (28).jpg]

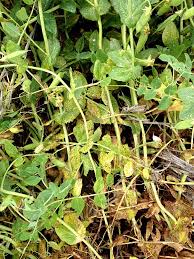

Supplement: S1 Dataset — (ZIP) [file pone.0307747.s001.zip › field pea dataset/Testing/Field_Pea_Ascochyta_Blight/Blight 30 (29).jpg]

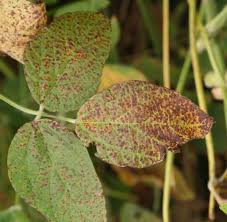

Supplement: S1 Dataset — (ZIP) [file pone.0307747.s001.zip › field pea dataset/Testing/Field_Pea_Ascochyta_Blight/Blight 30 (3).jpg]

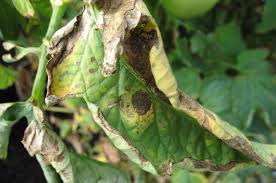

Supplement: S1 Dataset — (ZIP) [file pone.0307747.s001.zip › field pea dataset/Testing/Field_Pea_Ascochyta_Blight/Blight 30 (30).jpg]

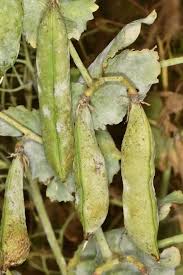

Supplement: S1 Dataset — (ZIP) [file pone.0307747.s001.zip › field pea dataset/Testing/Field_Pea_Ascochyta_Blight/Blight 30 (31).jpg]

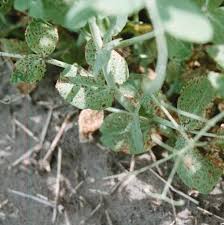

Supplement: S1 Dataset — (ZIP) [file pone.0307747.s001.zip › field pea dataset/Testing/Field_Pea_Ascochyta_Blight/Blight 30 (32).jpg]

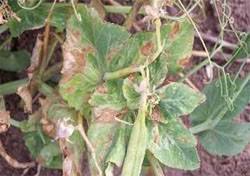

Supplement: S1 Dataset — (ZIP) [file pone.0307747.s001.zip › field pea dataset/Testing/Field_Pea_Ascochyta_Blight/Blight 30 (33).jpg]

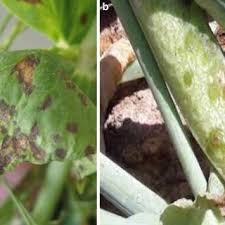

Supplement: S1 Dataset — (ZIP) [file pone.0307747.s001.zip › field pea dataset/Testing/Field_Pea_Ascochyta_Blight/Blight 30 (34).jpg]

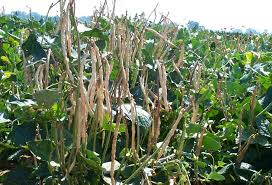

Supplement: S1 Dataset — (ZIP) [file pone.0307747.s001.zip › field pea dataset/Testing/Field_Pea_Ascochyta_Blight/Blight 30 (35).jpg]

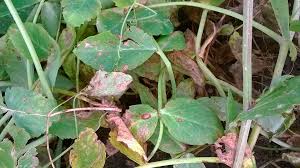

Supplement: S1 Dataset — (ZIP) [file pone.0307747.s001.zip › field pea dataset/Testing/Field_Pea_Ascochyta_Blight/Blight 30 (36).jpg]

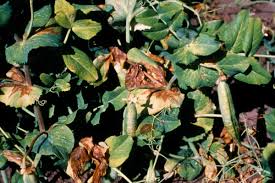

Supplement: S1 Dataset — (ZIP) [file pone.0307747.s001.zip › field pea dataset/Testing/Field_Pea_Ascochyta_Blight/Blight 30 (38).jpg]

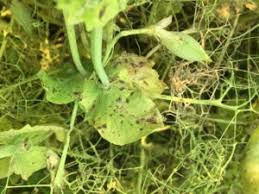

Supplement: S1 Dataset — (ZIP) [file pone.0307747.s001.zip › field pea dataset/Testing/Field_Pea_Ascochyta_Blight/Blight 30 (39).jpg]

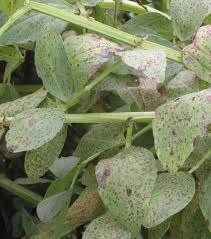

Supplement: S1 Dataset — (ZIP) [file pone.0307747.s001.zip › field pea dataset/Testing/Field_Pea_Ascochyta_Blight/Blight 30 (4).jpg]

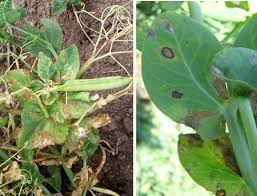

Supplement: S1 Dataset — (ZIP) [file pone.0307747.s001.zip › field pea dataset/Testing/Field_Pea_Ascochyta_Blight/Blight 30 (40).jpg]

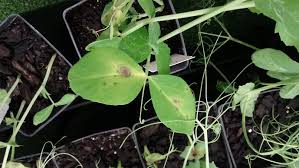

Supplement: S1 Dataset — (ZIP) [file pone.0307747.s001.zip › field pea dataset/Testing/Field_Pea_Ascochyta_Blight/Blight 30 (41).jpg]

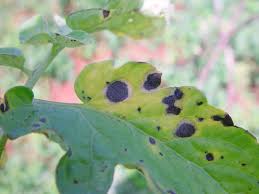

Supplement: S1 Dataset — (ZIP) [file pone.0307747.s001.zip › field pea dataset/Testing/Field_Pea_Ascochyta_Blight/Blight 30 (43).jpg]

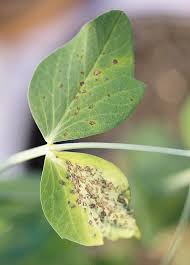

Supplement: S1 Dataset — (ZIP) [file pone.0307747.s001.zip › field pea dataset/Testing/Field_Pea_Ascochyta_Blight/Blight 30 (45).jpg]

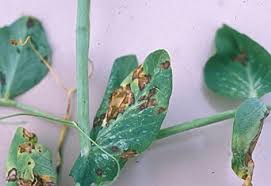

Supplement: S1 Dataset — (ZIP) [file pone.0307747.s001.zip › field pea dataset/Testing/Field_Pea_Ascochyta_Blight/Blight 30 (46).jpg]

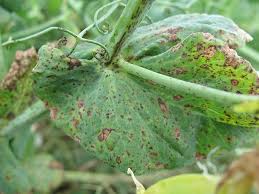

Supplement: S1 Dataset — (ZIP) [file pone.0307747.s001.zip › field pea dataset/Testing/Field_Pea_Ascochyta_Blight/Blight 30 (48).jpg]

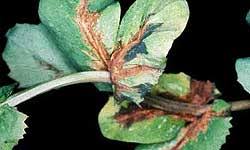

Supplement: S1 Dataset — (ZIP) [file pone.0307747.s001.zip › field pea dataset/Testing/Field_Pea_Ascochyta_Blight/Blight 30 (49).jpg]

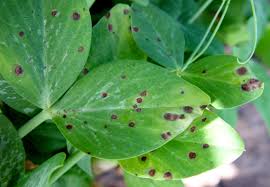

Supplement: S1 Dataset — (ZIP) [file pone.0307747.s001.zip › field pea dataset/Testing/Field_Pea_Ascochyta_Blight/Blight 30 (5).jpg]

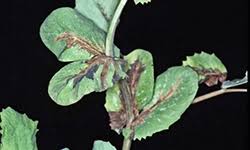

Supplement: S1 Dataset — (ZIP) [file pone.0307747.s001.zip › field pea dataset/Testing/Field_Pea_Ascochyta_Blight/Blight 30 (50).jpg]

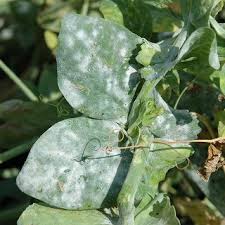

Supplement: S1 Dataset — (ZIP) [file pone.0307747.s001.zip › field pea dataset/Testing/Field_Pea_Ascochyta_Blight/Blight 30 (51).jpg]

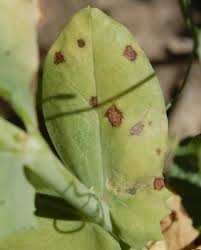

Supplement: S1 Dataset — (ZIP) [file pone.0307747.s001.zip › field pea dataset/Testing/Field_Pea_Ascochyta_Blight/Blight 30 (53).jpg]

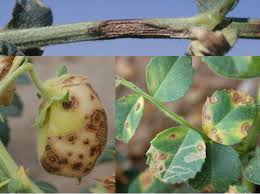

Supplement: S1 Dataset — (ZIP) [file pone.0307747.s001.zip › field pea dataset/Testing/Field_Pea_Ascochyta_Blight/Blight 30 (6).jpg]

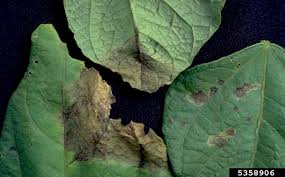

Supplement: S1 Dataset — (ZIP) [file pone.0307747.s001.zip › field pea dataset/Testing/Field_Pea_Ascochyta_Blight/Blight 30 (7).jpg]

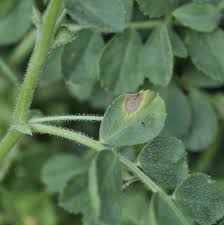

Supplement: S1 Dataset — (ZIP) [file pone.0307747.s001.zip › field pea dataset/Testing/Field_Pea_Ascochyta_Blight/Blight 30 (8).jpg]

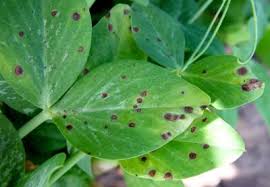

Supplement: S1 Dataset — (ZIP) [file pone.0307747.s001.zip › field pea dataset/Testing/Field_Pea_Ascochyta_Blight/Bright (1).jpg]

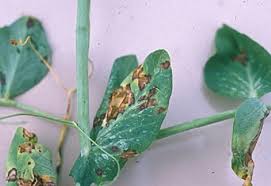

Supplement: S1 Dataset — (ZIP) [file pone.0307747.s001.zip › field pea dataset/Testing/Field_Pea_Ascochyta_Blight/Bright (2).jpg]

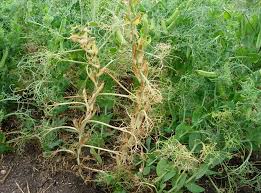

Supplement: S1 Dataset — (ZIP) [file pone.0307747.s001.zip › field pea dataset/Testing/Field_Pea_Ascochyta_Blight/Bright (3).jpg]

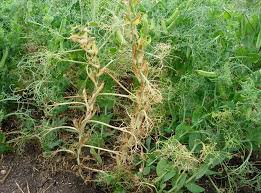

Supplement: S1 Dataset — (ZIP) [file pone.0307747.s001.zip › field pea dataset/Testing/Field_Pea_Ascochyta_Blight/Bright 11 (1).jpg]

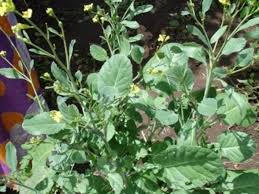

Supplement: S1 Dataset — (ZIP) [file pone.0307747.s001.zip › field pea dataset/Testing/Field_Pea_Healthy/Healthy 1011 (1).jpg]

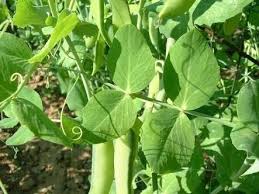

Supplement: S1 Dataset — (ZIP) [file pone.0307747.s001.zip › field pea dataset/Testing/Field_Pea_Healthy/Healthy 1011 (10).jpg]

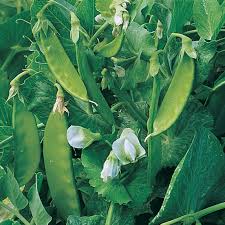

Supplement: S1 Dataset — (ZIP) [file pone.0307747.s001.zip › field pea dataset/Testing/Field_Pea_Healthy/Healthy 1011 (11).jpg]

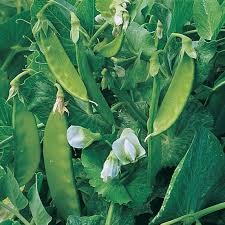

Supplement: S1 Dataset — (ZIP) [file pone.0307747.s001.zip › field pea dataset/Testing/Field_Pea_Healthy/Healthy 1011 (12).jpg]

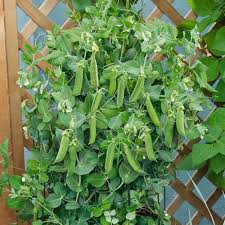

Supplement: S1 Dataset — (ZIP) [file pone.0307747.s001.zip › field pea dataset/Testing/Field_Pea_Healthy/Healthy 1011 (13).jpg]

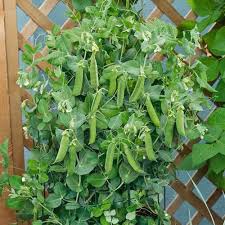

Supplement: S1 Dataset — (ZIP) [file pone.0307747.s001.zip › field pea dataset/Testing/Field_Pea_Healthy/Healthy 1011 (14).jpg]

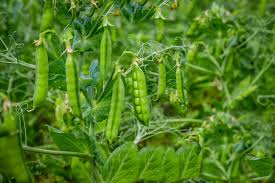

Supplement: S1 Dataset — (ZIP) [file pone.0307747.s001.zip › field pea dataset/Testing/Field_Pea_Healthy/Healthy 1011 (15).jpg]

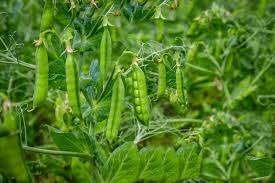

Supplement: S1 Dataset — (ZIP) [file pone.0307747.s001.zip › field pea dataset/Testing/Field_Pea_Healthy/Healthy 1011 (16).jpg]

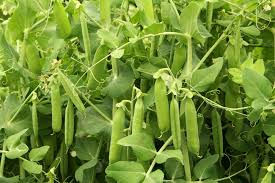

Supplement: S1 Dataset — (ZIP) [file pone.0307747.s001.zip › field pea dataset/Testing/Field_Pea_Healthy/Healthy 1011 (17).jpg]

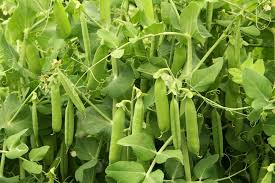

Supplement: S1 Dataset — (ZIP) [file pone.0307747.s001.zip › field pea dataset/Testing/Field_Pea_Healthy/Healthy 1011 (18).jpg]

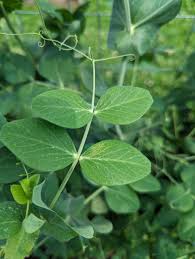

Supplement: S1 Dataset — (ZIP) [file pone.0307747.s001.zip › field pea dataset/Testing/Field_Pea_Healthy/Healthy 1011 (19).jpg]

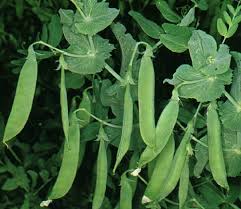

Supplement: S1 Dataset — (ZIP) [file pone.0307747.s001.zip › field pea dataset/Testing/Field_Pea_Healthy/Healthy 1011 (2).jpg]

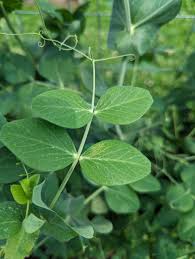

Supplement: S1 Dataset — (ZIP) [file pone.0307747.s001.zip › field pea dataset/Testing/Field_Pea_Healthy/Healthy 1011 (20).jpg]

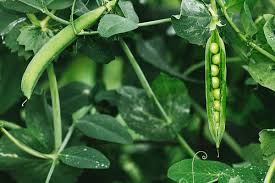

Supplement: S1 Dataset — (ZIP) [file pone.0307747.s001.zip › field pea dataset/Testing/Field_Pea_Healthy/Healthy 1011 (21).jpg]

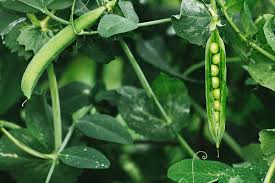

Supplement: S1 Dataset — (ZIP) [file pone.0307747.s001.zip › field pea dataset/Testing/Field_Pea_Healthy/Healthy 1011 (22).jpg]

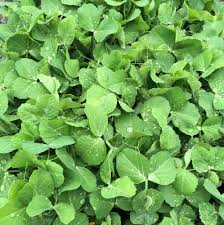

Supplement: S1 Dataset — (ZIP) [file pone.0307747.s001.zip › field pea dataset/Testing/Field_Pea_Healthy/Healthy 1011 (23).jpg]

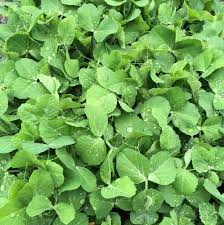

Supplement: S1 Dataset — (ZIP) [file pone.0307747.s001.zip › field pea dataset/Testing/Field_Pea_Healthy/Healthy 1011 (24).jpg]

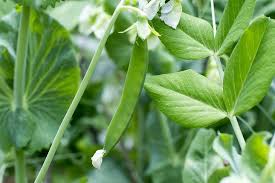

Supplement: S1 Dataset — (ZIP) [file pone.0307747.s001.zip › field pea dataset/Testing/Field_Pea_Healthy/Healthy 1011 (25).jpg]

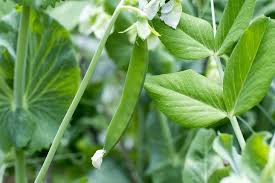

Supplement: S1 Dataset — (ZIP) [file pone.0307747.s001.zip › field pea dataset/Testing/Field_Pea_Healthy/Healthy 1011 (26).jpg]

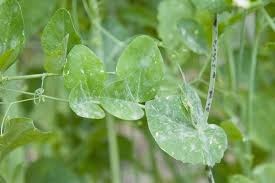

Supplement: S1 Dataset — (ZIP) [file pone.0307747.s001.zip › field pea dataset/Testing/Field_Pea_Healthy/Healthy 1011 (27).jpg]

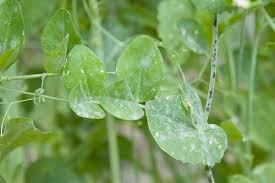

Supplement: S1 Dataset — (ZIP) [file pone.0307747.s001.zip › field pea dataset/Testing/Field_Pea_Healthy/Healthy 1011 (28).jpg]

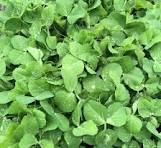

Supplement: S1 Dataset — (ZIP) [file pone.0307747.s001.zip › field pea dataset/Testing/Field_Pea_Healthy/Healthy 1011 (29).jpg]

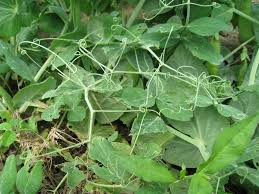

Supplement: S1 Dataset — (ZIP) [file pone.0307747.s001.zip › field pea dataset/Testing/Field_Pea_Healthy/Healthy 1011 (3).jpg]

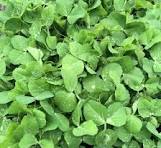

Supplement: S1 Dataset — (ZIP) [file pone.0307747.s001.zip › field pea dataset/Testing/Field_Pea_Healthy/Healthy 1011 (30).jpg]

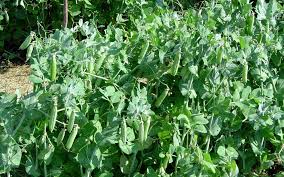

Supplement: S1 Dataset — (ZIP) [file pone.0307747.s001.zip › field pea dataset/Testing/Field_Pea_Healthy/Healthy 1011 (31).jpg]

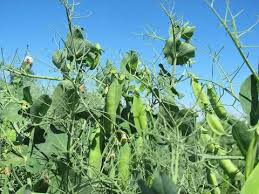

Supplement: S1 Dataset — (ZIP) [file pone.0307747.s001.zip › field pea dataset/Testing/Field_Pea_Healthy/Healthy 1011 (32).jpg]

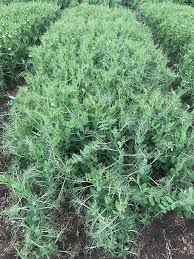

Supplement: S1 Dataset — (ZIP) [file pone.0307747.s001.zip › field pea dataset/Testing/Field_Pea_Healthy/Healthy 1011 (33).jpg]

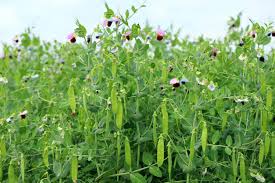

Supplement: S1 Dataset — (ZIP) [file pone.0307747.s001.zip › field pea dataset/Testing/Field_Pea_Healthy/Healthy 1011 (34).jpg]

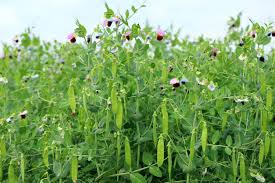

Supplement: S1 Dataset — (ZIP) [file pone.0307747.s001.zip › field pea dataset/Testing/Field_Pea_Healthy/Healthy 1011 (35).jpg]

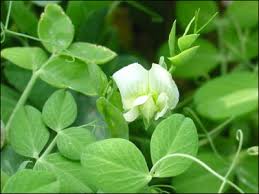

Supplement: S1 Dataset — (ZIP) [file pone.0307747.s001.zip › field pea dataset/Testing/Field_Pea_Healthy/Healthy 1011 (36).jpg]

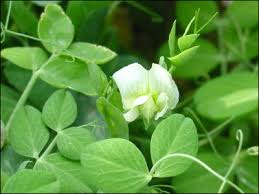

Supplement: S1 Dataset — (ZIP) [file pone.0307747.s001.zip › field pea dataset/Testing/Field_Pea_Healthy/Healthy 1011 (37).jpg]

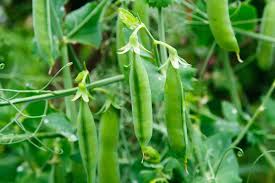

Supplement: S1 Dataset — (ZIP) [file pone.0307747.s001.zip › field pea dataset/Testing/Field_Pea_Healthy/Healthy 1011 (38).jpg]

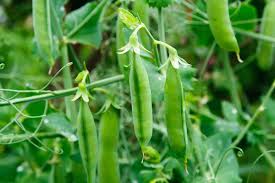

Supplement: S1 Dataset — (ZIP) [file pone.0307747.s001.zip › field pea dataset/Testing/Field_Pea_Healthy/Healthy 1011 (39).jpg]

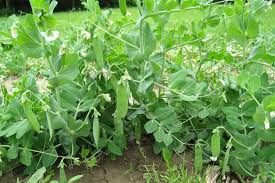

Supplement: S1 Dataset — (ZIP) [file pone.0307747.s001.zip › field pea dataset/Testing/Field_Pea_Healthy/Healthy 1011 (4).jpg]

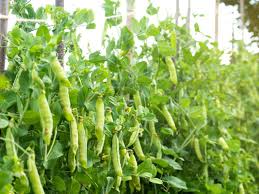

Supplement: S1 Dataset — (ZIP) [file pone.0307747.s001.zip › field pea dataset/Testing/Field_Pea_Healthy/Healthy 1011 (40).jpg]

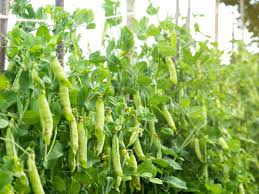

Supplement: S1 Dataset — (ZIP) [file pone.0307747.s001.zip › field pea dataset/Testing/Field_Pea_Healthy/Healthy 1011 (41).jpg]

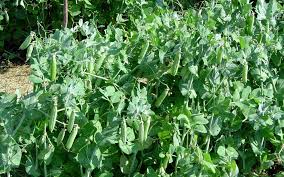

Supplement: S1 Dataset — (ZIP) [file pone.0307747.s001.zip › field pea dataset/Testing/Field_Pea_Healthy/Healthy 1011 (42).jpg]

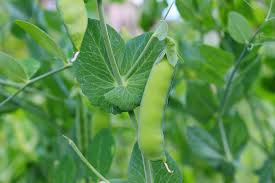

Supplement: S1 Dataset — (ZIP) [file pone.0307747.s001.zip › field pea dataset/Testing/Field_Pea_Healthy/Healthy 1011 (44).jpg]

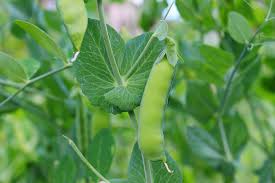

Supplement: S1 Dataset — (ZIP) [file pone.0307747.s001.zip › field pea dataset/Testing/Field_Pea_Healthy/Healthy 1011 (45).jpg]

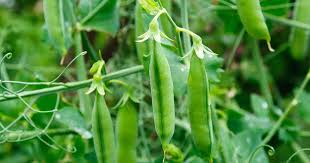

Supplement: S1 Dataset — (ZIP) [file pone.0307747.s001.zip › field pea dataset/Testing/Field_Pea_Healthy/Healthy 1011 (46).jpg]

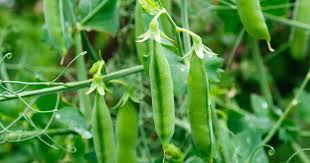

Supplement: S1 Dataset — (ZIP) [file pone.0307747.s001.zip › field pea dataset/Testing/Field_Pea_Healthy/Healthy 1011 (47).jpg]

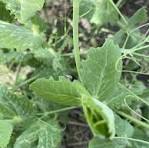

Supplement: S1 Dataset — (ZIP) [file pone.0307747.s001.zip › field pea dataset/Testing/Field_Pea_Healthy/Healthy 1011 (48).jpg]

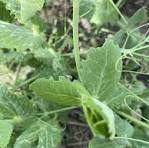

Supplement: S1 Dataset — (ZIP) [file pone.0307747.s001.zip › field pea dataset/Testing/Field_Pea_Healthy/Healthy 1011 (49).jpg]

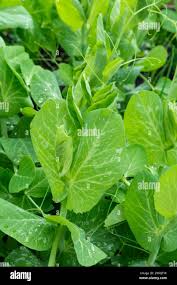

Supplement: S1 Dataset — (ZIP) [file pone.0307747.s001.zip › field pea dataset/Testing/Field_Pea_Healthy/Healthy 1011 (5).jpg]

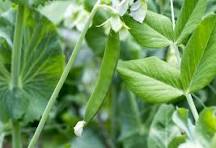

Supplement: S1 Dataset — (ZIP) [file pone.0307747.s001.zip › field pea dataset/Testing/Field_Pea_Healthy/Healthy 1011 (50).jpg]

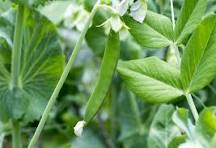

Supplement: S1 Dataset — (ZIP) [file pone.0307747.s001.zip › field pea dataset/Testing/Field_Pea_Healthy/Healthy 1011 (51).jpg]

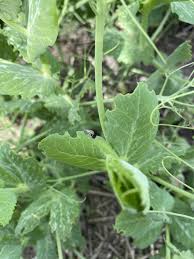

Supplement: S1 Dataset — (ZIP) [file pone.0307747.s001.zip › field pea dataset/Testing/Field_Pea_Healthy/Healthy 1011 (52).jpg]

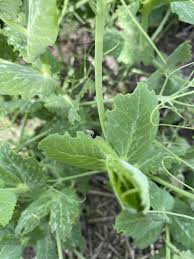

Supplement: S1 Dataset — (ZIP) [file pone.0307747.s001.zip › field pea dataset/Testing/Field_Pea_Healthy/Healthy 1011 (53).jpg]

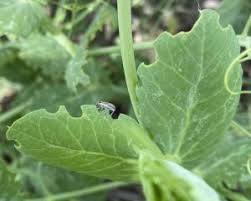

Supplement: S1 Dataset — (ZIP) [file pone.0307747.s001.zip › field pea dataset/Testing/Field_Pea_Healthy/Healthy 1011 (54).jpg]
