# Supplementary material for: Field pea leaf disease classification using a deep learning approach
Source: PLoS One. 2024 Jul 25;19(7):e0307747. doi: 10.1371/journal.pone.0307747 (PMC11271925; doi:10.1371/journal.pone.0307747)
Supplement: Attachment — Submitted filename: PONE-D-24-10799.docx [file pone.0307747.s002.docx]

**Field Pea Leaf Disease** 1 **Classification Using a Deep Learning Approach**

**Comments**

**Title:** Appropriate

**Abstract:**

You have not indicated the significance of your study, which is usually the last statement in the abstract section.

**Introduction:**

Line 35: ‘Field peas are grown around the world….’ This has already been indicated, so there is no need to repeat it. The next statement ‘…and have 86% loss due to powdery mildew disease’ is contradictory to what you alluded to earlier that ‘…...these diseases can reduce yields by 20–30%’ (line 31). I suggest that you amalgamate both sentences and have a unified statement offering the specific statistics with a relevant citation.

**Related works:**

Line 63: ‘The PlantVillage dataset was used…’ Provide a link to indicate the dataset used. The same should be applied for line 78: ‘A database with 5,000 images of potatoes was employed.’ Line 93: ‘…on the 1,33 images of the test set.’ Is it really 133 images?

I don’t understand why you indicated the contributions of your work in the related works section before providing the methods and results obtained. This would have been appropriate for the conclusions of your study.

**Methods and materials:**

Shouldn’t it be materials and methods? You need to indicate the materials used in the study. Generally, this section has provided limited information on the protocol you used. For example, line 148: ‘we labeled the images of field peas…’ How many images? How were they obtained? Which camera was used to capture the images? Where was the study conducted?

Line 151: ‘we employed several approaches, such as…’ Please specify the precise approaches used in the study.

Line 154: ‘models were tested using the test images…’ How many images? Where were they obtained? Did you use a specific field pea cultivar?

**Results and discussion:**

The information lacking in the methods section is provided in the results section, which is not appropriate. This section should only provide the results you obtained from the study and not how the study was conducted. I have also not seen any discussion of your results in comparison to previous studies. Generally, you have provided limited/incomplete information in the methods, results, and discussion sections.
